# Supplementary material for: FERN – a Java framework for stochastic simulation and evaluation of reaction networks
Source: BMC Bioinformatics. 2008 Aug 29;9:356. doi: 10.1186/1471-2105-9-356 (PMC2553347; doi:10.1186/1471-2105-9-356)
Supplement: Additional file 1 — FERN distribution, Version 1.3. This archive contains the FERN source code and binaries as well as documentation and example models in FernML and SBML. [file 1471-2105-9-356-S1.zip › fern/doc/javadoc/fern/simulation/algorithm/class-use/GillespieEnhanced.html]

Uses of Class fern.simulation.algorithm.GillespieEnhanced


---


|  |  |  |
| --- | --- | --- |
| **Overview** \| **Package** \| **Class** \| **Use** \| **Tree** \| **Deprecated** \| **Index** \| **Help** | |  |
| PREV   NEXT | **FRAMES**    **NO FRAMES**     **All Classes** |  |


---


## **Uses of Class fern.simulation.algorithm.GillespieEnhanced**

| Packages that use GillespieEnhanced | |
| --- | --- |
| **fern.simulation.algorithm** | Provides algorithms for stochastic simultion of reaction networks. |

Uses of GillespieEnhanced in fern.simulation.algorithm

| Subclasses of GillespieEnhanced in fern.simulation.algorithm | |
| --- | --- |
| `class` | `AbstractBaseTauLeaping`             Base class for all tau leaping procedures (which are different in the methods choosing the timestep candidates for critical and noncritical reactions). |
| `class` | `AbstractTauLeapingPropensityBoundSimulator`             There are some possibilities to bind the expected change of the propensities by a value epsilon in order to fulfill the leap condition. |
| `class` | `TauLeapingAbsoluteBoundSimulator`             There are some possibilities to bind the expected change of the propensities by a value epsilon in order to fulfill the leap condition. |
| `class` | `TauLeapingRelativeBoundSimulator`             There are some possibilities to bind the expected change of the propensities by a value epsilon in order to fulfill the leap condition. |
| `class` | `TauLeapingSpeciesPopulationBoundSimulator`             There are some possibilities to bind the expected change of the propensities by a value epsilon in order to fulfill the leap condition. |

---


|  |  |  |
| --- | --- | --- |
| **Overview** \| **Package** \| **Class** \| **Use** \| **Tree** \| **Deprecated** \| **Index** \| **Help** | |  |
| PREV   NEXT | **FRAMES**    **NO FRAMES**     **All Classes** |  |


---
